# Microbial composition play the leading role in volatile fatty acid production in the fermentation of different scale of corn stover with rumen fluid

**DOI:** 10.3389/fbioe.2023.1275454

**Published:** 2024-01-04

**Authors:** Haiyan Zhang, Wanqin Zhang, Shunli Wang, Zhiping Zhu, Hongmin Dong

**Affiliations:** ^1^ Institute of Environment and Sustainable Development in Agriculture, Chinese Academy of Agricultural Sciences, Beijing, China; ^2^ China Huadian Engineering Co., Ltd., Beijing, China

**Keywords:** corn stover, fermentation, microorganisms, substrate structure, volatile fatty acid

## Abstract

Rumen fluid is a natural and green biocatalyst that can efficiently degrade biomass into volatile fatty acid (VFA) used to produce value-added materials. But the essence of high degradation efficiency in the rumen has not been fully analyzed. This study investigated the contribution of substrate structure and microbial composition to volatile fatty acid production in the fermentation of corn stover. The ball milled corn stover were innovatively applied to ferment with the rumen fluid collected at different digestion times. Exogeneous cellulase was also added to the ruminal fermentation to further reveal the inner mechanism. With prolonged digestion time, the microbial community relative abundance levels of *Bacteroidetes* and *Firmicutes* increased from 29.98% to 72.74% and decreased from 51.76% to 22.11%, respectively. The highest VFA production of the corn stover was achieved via treatment with the rumen fluid collected at 24 h which was up to 9508 mg/L. The ball milled corn stover achieved high VFA production because of the more accessible substrate structure. The application of exogenous cellulase has no significant influence to the ruminal fermentation. The microbial community abundance contributed more to the VFA production compared with the substrate structures.

## 1 Introduction

Lignocellulosic biomass is a common and abundantly available resource with great potential for conversion into high-valued biofuels and biomaterials (Shen et al., 2020). However, the utilization of lignocellulosic biomass for bioconversion is challenging because cellulose, hemicellulose, and lignin are intertwined to form a rigid and complex structure resistant to degradation. To improve the degradation efficiency, different pretreatments have been studied such as physical, chemical, biological and combinations (Zhang et al., 2021). However, to develop a green and cost-effective method is still necessary to overcome the high investment and possible pollution.

Rumen microorganisms, characterized by high hydrolysis and acidification efficiency, have been recognized as the efficient natural biological system to degrade biomass materials according to extensive relevant studies worldwide (Seshadri et al., 2018; Xing et al., 2020b). Volatile fatty acids (VFAs) are the major intermediate products in ruminal fermentation and could be used to produce other products such as hydrogen and bioplastics (Ebrahimian et al., 2020). The VFAs converted from the low-cost feedstocks could improve the economic sustainability of industrial polyhydroxyalkanoates production (Zhou et al., 2023). Considerable value can be achieved by maximizing use of the natural microbial community and applying it to practical production (Liang et al., 2020). In recent years, rumen fluid has been directly used as an inoculum in the anaerobic digestion to improve the fermentation efficiency of lignocellulosic biomass (Xing et al., 2020a). The research mainly focuses on improving the fermentation efficiency by optimizing the reaction conditions, such as pH, solid-liquid ratio, and preservation method (Wang et al., 2018; Takizawa et al., 2020; Njokweni et al., 2021). However, due to the complexity of the rumen microorganisms and their selectivity to the environment, the essence of high degradation efficiency in the rumen has not been fully analyzed.

In addition to environmental impacts to the ruminal fermentation, characteristics of the substrate and the rumen fluid itself may also be important factors. Ball milling pretreatment is an effective pretreatment method of decreasing the particle size to cellular scale and promoting the hydrolysis efficiency of biomass significantly which was widely studied (Zhang et al., 2016a; Wu et al., 2021). However, the study on the contribution of ball milling to ruminal fermentation and the influence of physicochemical properties of the substrate on fermentation performance with ruminal microorganisms is scarce.

In the fermentation process, the microbial composition is one of the key parameters to the degradation rate. Some researchers have reported numerous cellulase and xylanase existing in rumen fluid, synergistically promoting the degradation efficiency (Hess et al., 2011; Solomon et al., 2016). With extension of the digestion time, relative abundance of microorganisms in rumen may change dynamically. Knowledge regarding the kinetic changes of the microbiota with the biomass digestion in the rumen is considerably limited. In addition, no reports are available on investigating how the rumen microorganisms change and affect the biomass degradation characteristics with the digestion time. Investigating the effects of substrate structure and ruminal composition to the degradation of biomass contributes to the efficient usage of the microorganisms thus helping reduce the degradation cost.

The objective of this study is to determine the relative contribution of substrate structure and microbial community to VFA production in the ruminal fermentation. The dynamic variation of microbial community of the rumen fluid collected at different digestion times and the corresponding degradation characteristics of corn stover were comparatively analyzed. Ball milling pretreatment was also innovatively applied to investigate the influence of substrate structure on the hydrolysis properties. Additionally, the relationship between microbial abundance and hydrolysis yield was quantified to illustrate the dependency mechanism of VFA production on rumen microorganisms. This study will shed lights on improving the ruminal degradation rate of corn stover, and provide some references to broaden the application of ruminal microorganisms.

## 2 Materials and methods

### 2.1 Preparation of corn stover

The corn stover (leftovers after harvesting) used in all experiments were collected from Shangzhuang experimental farm of China Agricultural University. The collected corn stover was air-dried and chopped into small particles (approximately 1–2 cm). The chopped materials were further milled with an RT-34 hammer mill and sieved through a 40-mesh screen to obtain the coarse milled (CM) samples. Then, 150 g of CM samples were further ground with ZrO_2_ balls with a 1:2 volume ratio for 30 min using an ultrafine vibration grinding mill to obtain the ball-milled (BM) samples. All prepared samples were stored in sealed bags at room temperature for further use.

### 2.2 Collection of the rumen fluid

Rumen fluid used in all experiments was withdrawn from the Nankou experimental farm of the Chinese Academy of Agricultural Sciences. Four healthy adult Du Han disease-free hybrid mutton sheep of similar weight (about 36 kg) having installed permanent rumen fistula were chosen as the rumen fluid donor. The rumen fluids were collected at 3, 6, 12, 24, 36 and 48 h after feeding. The collected fluids from different sheep were mixed and filtered with four layers of gauze to ensure uniformity. The fluids were then quickly transferred to pre-warmed (39 C) thermos flasks for future use in 6 h. The animal procedures used in this study were reviewed and approved by the Animal Ethics Committee of the Chinese Academy of Agricultural Sciences (protocol number 057-2019).

### 2.3 Characterization of the corn stover

The cellulose, hemicellulose, and lignin contents of the corn stover were measured according to the technical method proposed by National Renewable Energy Laboratory (NREL) (NREL/TP-510-42618) (Sluiter et al., 2008). First, 300 mg of the CM and BM samples were put into pressure tubes, respectively, before adding 3 mL of 72% w/w sulfuric acid. Then, the mixtures were incubated at 30 C for 60 min in a water bath, where the samples were stirred every 5–10 min to facilitate complete hydrolysis. Afterward, 84 mL of deionized water was added to the tubes to dilute the acid concentration to 4%, and autoclaved at 121°C for 60 min. The supernatants were filtered upon completion of the hydrolysis and the sugar concentrations were determined with a high-performance liquid chromatography (HPLC) system to calculate the cellulose and hemicellulose contents. Meanwhile, the hydrolysis residues were dried and burned to calculate the acid-insoluble lignin content. The acid-soluble lignin was calculated by determining the supernatant absorbance at 320 nm with a UV-visible spectrophotometer.

The particle size distributions of the CM and BM corn stover were measured using a Mastersizer 3000 laser diffraction particle size analyzer set at the wet mode. The samples were dispersed with distilled water into the measurement instrument with an ultrasonic intensity of 10% and stirred at 2000 rpm.

The surface morphological properties of the CM and BM corn stover were characterized with a Hitachi SU8010 high-resolution scanning electron microscope (SEM) instrument. The specimens were Pt-plated to obtain clear images with the accelerating voltage set at 3.0 kV.

Cellulose crystallinity was measured using a Bruker D8 advance X-ray diffractometer. The measurement mode was set with a scanning range of 5°–40° and a step size of 0.2°. The crystallinity index (CrI) was calculated according to the formula proposed by the previous researcher (Segal et al., 1959), which is expressed as *CrI =* (*I*_*002*_ *–I*_*am*_)*/I*_*002*_ × *100%. I*_*002*_ is the maximum intensity of the crystalline diffraction peak near 2θ = 22°, and *I*_*am*_ referred to the amorphous diffraction intensity near 2θ = 18°.

The specific surface area and pore volume of the CM and BM samples were measured using a MicroActive ASAP 2460 physical adsorption analyzer. The specific surface area was calculated based on the Brunauer–Emmett–Teller (BET) model (Barrett et al., 1951), and the pore volume was estimated via the Barrett–Joyner–Halenda (BJH) model (Brunauer et al., 1938).

### 2.4 Determining microbial diversity in rumen fluid

As described in the “Section 2.2”, the rumen fluids were collected at 3, 6, 12, 24, 36 and 48 h after feeding for analysis of microbial diversity. Microorganisms in the rumen fluids were investigated by using a high-throughput sequencing technique. The DNA was extracted from the samples and amplified via PCR using the primers 338F (5′-ACT​CCT​ACG​GGA​GGC​AGC​A-3′) and 806R (5′-GGACTACHVGGGTWTCTAAT-3′) in the V3-V4 hypervariable regions of 16S rRNA. The PCR products were identified, purified, and sequenced by the Illumina Miseq PE300/NovaSeq PE250 platform. The sequence annotation classification of each species was analyzed based on the Silva 16s rRNA database (v138) using the RDP classifier (Wang et al., 2007).

### 2.5 Assessing fermentation of corn stover

As described in the “Section 2.2”, the rumen fluids collected at 6, 24, and 48 h were chosen to treat the corn stover. The experiments were conducted in 150 mL conical flasks with 5 g samples and 100 mL rumen fluid set at a 5% (w/v) solid loading. All conical flasks were sealed with plastic wraps and incubated at 39 C in an incubator and shaken at 150 rpm. The incubation was conducted for 4, 12, 24, 48, 72, 96, and 120 h, respectively. The treatment conditions were chosen according to the previous studies (Zhang et al., 2016b; Li et al., 2017). Each reaction was performed in duplicate, and substrate blanks and rumen fluid blanks were also conducted as the control to correct the results. After incubation, 5 mL of mixtures are taken, and the solid and liquid are separated by centrifuging the mixture at 8,000 rpm for 5 min. The supernatants were then filtered with a 0.22 μm filter to analyze the VFA content. The VFA contents were determined by Agilent GC 7890A gas chromatography with a flame ionization detector equipped with a DB-FFAP capillary column (30 m × 0.32 mm × 0.25 µm). Nitrogen was set as the carrier gas with a flow rate of 30 mL/min. The temperatures of the injection port and the detector were set at 250 C and 300 C, respectively.

### 2.6 Application of exogeneous cellulase to treat the corn stover with rumen fluid

The exogeneous cellulase (Cellic CTec2) used in the ruminal fermentation is purchased from Sigma-Aldrich (St. Louis, MO, United States). The 10 FPU/(g dry substance) enzyme loading was optimized in previous enzymatic hydrolysis experiments (Zhang et al., 2018). All hydrolysis conditions were the same as those of the experiments in the above ruminal fermentation (Sub-Section 2.5). The VFA and glucose contents in the supernate were measured after hydrolysis. The glucose yields were analyzed based on the standard method of NREL (NREL/TP-510-42623) using an HPLC system equipped with a Bio-Rad HPX-87H column (Sluiter et al., 2008). The mobile phase was 0.005M H_2_SO_4_ with a flow rate of 0.6 mL/min at the column temperature of 55 C and the detection time for 30 min.

### 2.7 Statistical analysis

Error bars were derived from the standard deviation of panel data. All the data were analyzed using Microsoft Excel 2019 for Windows. The figures were plotted and analyzed in Origin 8.5.

## 3 Results and discussion

### 3.1 Initial characteristics of corn stover

The cellulosic composition and structural properties of the prepared corn stover are described in Table 1. The average particle sizes of CM and BM corn stover were 240.5 μm and 18.4 μm, respectively. The particle size of BM corn stover distinctively decreased and distributed more uniformly, which could improve the rheological behavior with reduced slurry viscosity and improved hydrolysis yield (Lu et al., 2020). The specific surface area and pore volume of BM corn stover were approximately 2.5-folds higher than CM corn stover. According to a previous study, holes in the fiber might work as channelling for rumen microorganisms to degrade the fibers (Yue et al., 2013). The accessible structural properties of BM corn stover would benefit adsorption of the microorganisms to substrate and facilitate the hydrolysis. The cellulose crystallinity of BM corn stover decreased by 33.42% than CM corn stover, which facilitated the hydrolysis. All these changes indicated that ultrafine grinding could substantially destroy fiber structure of the lignocellulose.

**TABLE 1:** Cellulosic composition and structural properties of the corn stover.

Sample	Particle size (μm)	Specific surface area (m^2^/g)	Pore volume (cm^3^/g)	Cellulose crystallinity index (%)	Cellulose (%)	Hemicellulose (%)	Lignin (%)
Coarse milled sample (CM)	240.5 ± 3.54	0.67	0.0016	48.38	34.70 ± 0.29	14.64 ± 0.16	21.76 ± 1.76
Ball-milled sample (BM)	18.4 ± 0.14	1.67	0.0041	32.21	32.90 ± 0.21	15.35 ± 0.28	22.49 ± 0.42

**FIGURE 1:**
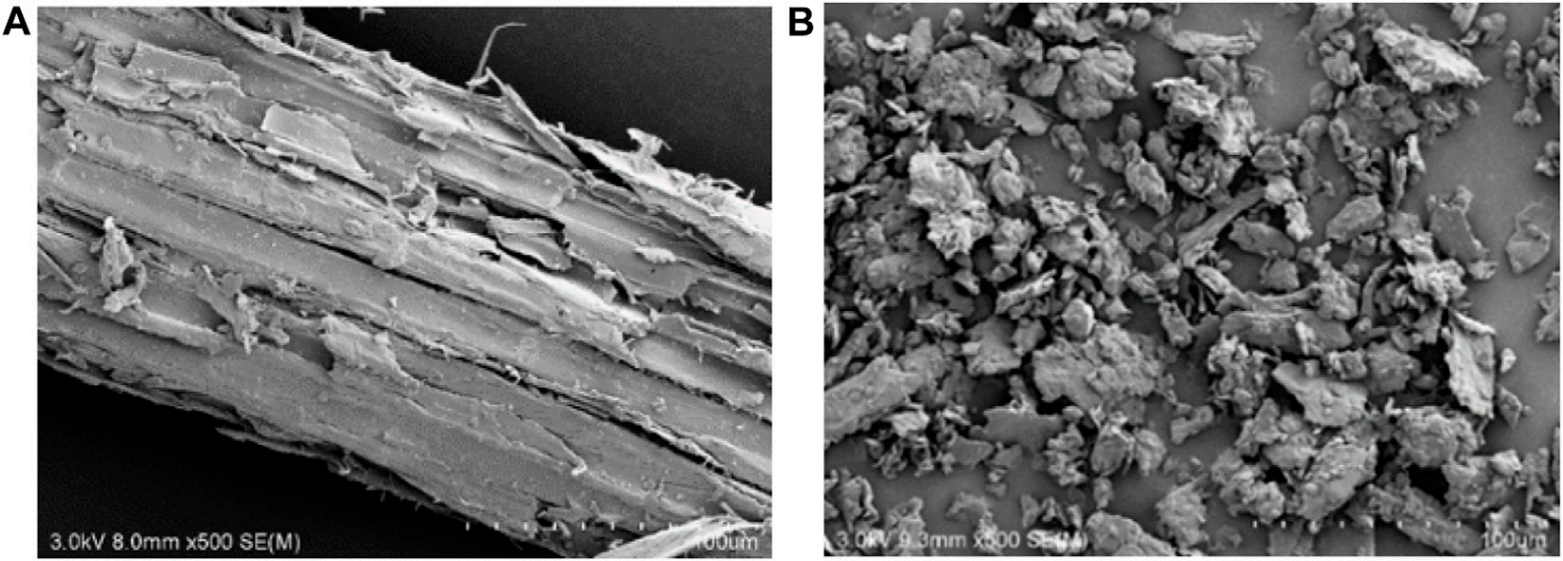
SEM observations of coarse milled (CM) **(A)** and ball-milled (BM) **(B)** corn stover.

According to the SEM observations of the corn stover (Figure 1), the fiber bundles of BM samples could hardly be seen. The surface of BM samples was rough, and small particles may have gathered to show agglomeration phenomena (Ji et al., 2016). On the contrary, the structure of CM samples was relatively intact, and fiber bundles were found on CM samples. The apparent structure changes of BM corn stover may facilitate ruminal microbial colonization and fiber digestion (Zhao et al., 2018).

Despite considerable differences in structural properties and surface morphology, the carbohydrates and lignin content in both CM and BM samples showed no significant difference (Table 1). These results indicated that ball milling pretreatment could only change the structure without impacting the substrate composition which is consistent with previous study (Chen et al., 2016). Even though the total chemical composition of BM corn stover was not changed, the surface composition could be redistributed. Moreover, the inner cellulose would be exposed to the surface, which may facilitate the hydrolysis (Zhang et al., 2017).

### 3.2 Initial characteristics of the rumen fluid

Based on the previous report, the initial characteristics of rumen fluid could provide some basic information for fermentation experiments (Ribeiro et al., 2017). The VFA concentration and pH of the initial rumen fluid collected at different digestion times are shown in Table 2. The dominant VFA were acetic acid, followed by propionic and butyric acid. The VFA concentrations decreased with the prolonged digestion time due to the diurnal dynamics in rumen (van Lingen et al., 2017). The products in ruminal fermentation were mainly short-chain fatty acids, which are easy to be used and more conducive for effective and prominent hydrogen recovery (Wang et al., 2020). The pH of rumen fluid slightly increased ranging from 6 to 7.5 with digestion time, indicating that the biomass fermentation in the rumen worked under neutral condition.

**TABLE 2:** Initial characteristics of the rumen fluid collected at different digestion times.

Collecting time	3 h	6 h	12 h	24 h	36 h	48 h
pH	6.02 ± 0.41	6.19 ± 0.19	6.35 ± 0.08	7.10 ± 0.08	7.08 ± 0.16	7.32 ± 0.12
Acetic acid (mg/L)	4485.51 ± 835.40	3836.79 ± 289.45	2873.42 ± 244.63	994.42 ± 308.71	603.97 ± 511.09	204.48 ± 183.10
Propionic acid (mg/L)	1583.46 ± 338.16	1168.65 ± 342.99	829.91 ± 257.76	262.74 ± 102.95	213.03 ± 155.94	91.31 ± 51.77
Butyric acid (mg/L)	1088.42 ± 24.58	1091.16 ± 80.66	659.65 ± 356.30	304.44 ± 78.88	187.60 ± 88.91	116.73 ± 35.26
Volatile fatty acids (VFA) concentration (mg/L)	7157.39 ± 1131.27	6096.59 ± 554.96	4362.98 ± 396.33	1561.61 ± 448.92	1004.59 ± 752.51	412.53 ± 266.17

### 3.3 Analysis of rumen microorganisms

**FIGURE 2:**
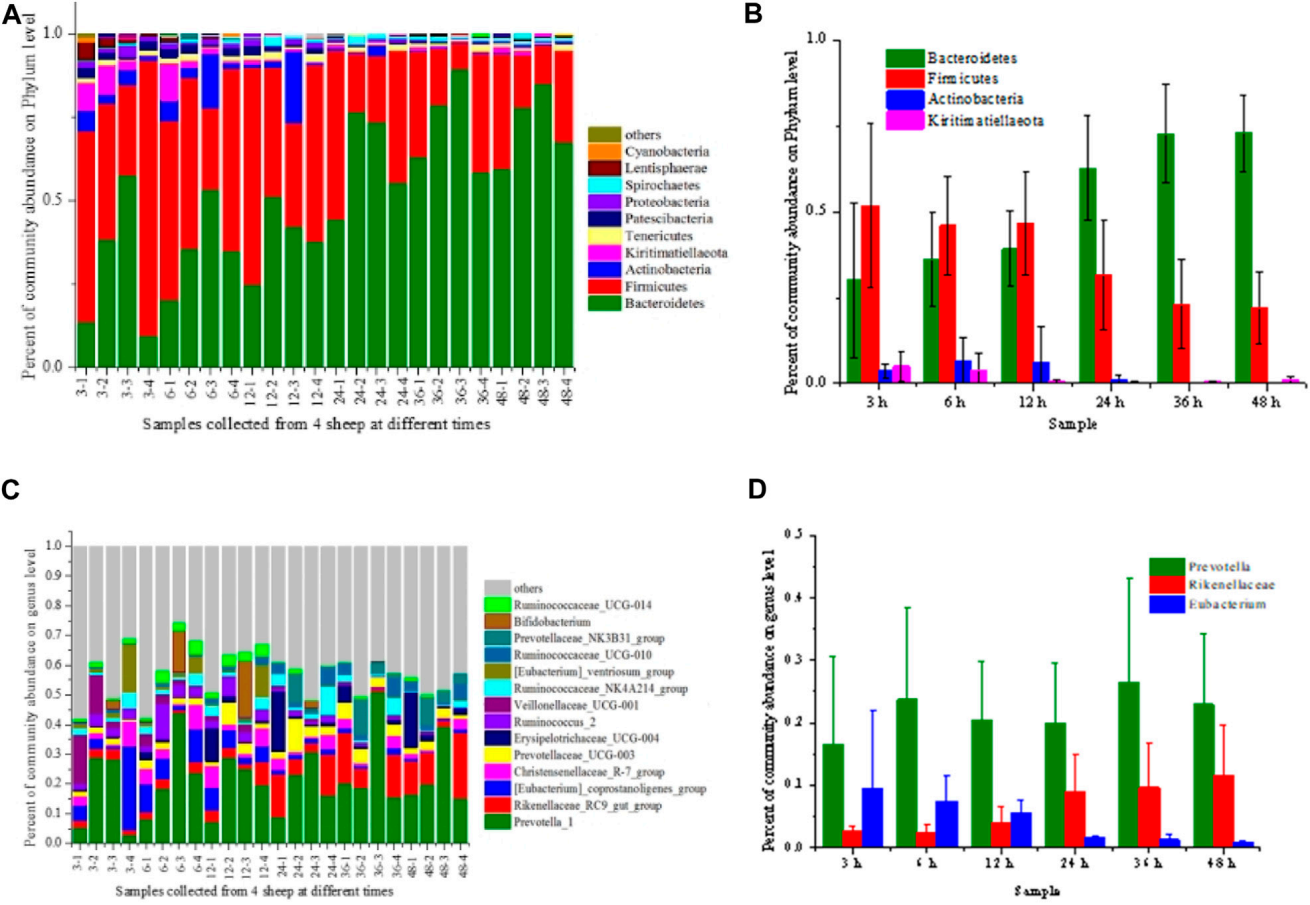
Relative abundance of the microbial community at the phylum level **(A, B)** and genus level **(C, D)**. **(A, C)** represented the microbial community profile collected from 4 different sheep (labelled as −1, −2, −3 and −4, respectively), and **(B, D)** represented the mean value of the relative abundance of the dominant microbes from 4 different sheep. Others in the legend referred to the microbial abundance lower than 0.01.

Rumen microorganisms are crucial in degradation of the lignocellulosic biomass, where different kinds of microbials synergistically participate in the hydrolysis (Stewart et al., 2018). Figure 2 shows the dynamic characteristics of the rumen microorganisms at different digestion times. At the phylum level (Figure 2A, B), *Bacteroidetes* and *Firmicutes* were the most abundant microorganisms and showed high standard deviation, accounting for 81.75%–94.85% of the microbial communities. Xin et al. also reported that *Bacteroidetes* and *Firmicutes* were the dominant bacteria for different breeds in various regions (Xin et al., 2019). With the prolonged digestion time, the abundance levels of *Bacteroidetes* increased from 29.98% to 72.74%, while *Firmicutes* decreased from 51.76% to 22.11%. Henderson et al. reported that *Firmicutes* and *Bacteroidetes* are predominant in the rumen (Henderson et al., 2015) and contributed 68% and 12.8% of Hungate genome sequences, respectively (Seshadri et al., 2018). Stewart et al. demonstrated that *Bacteroidetes* and *Firmicutes* together contributed the largest number of bacteria group. However, 5.7% of the proteome of *Bacteroidetes* was devoted to CAZyme activity while 3.2% in *Firmicutes* (Stewart et al., 2019). Carbohydrate metabolism was first attributed to high-cellulolytic activity and high fiber attachment. At beginning of the digestion, the abundance of *Bacteroidetes* was higher than that of *Firmicutes.* With the degradation of the cellulose, the carbohydrate composition decreased, leading to the increase in *Bacteroidetes* and the decrease in *Firmicutes*. The *Actinobacteria* and *Kiritimatiellaeota* were relatively high after feeding for 12 h but can barely be detected after 24 h. These kinetic changes of community abundance of the microbials in the rumen might be related to pH and acidic conditions as they could influence the hydrolysis properties of the substrate (van Lingen et al., 2017).

The microbes were complex at the genus level (Figure 2C, D) with *Prevotella*, *Rikenellaceae* and *Eubacterium* were the most abundant genera, presenting an apparent kinetic trend. The relative abundance of *Prevotella* showed a fluctuant variation from 16.48% to 26.56%, which increased at 6 h but slightly decreased after 12 h and then increased again at 36 h. *Prevotella* was the dominant cellulolytic genera in the rumen of various ruminants, producing a wide range of enzymes to degrade the cellulose and hemicellulose (Palevich et al., 2019; Nguyen et al., 2020). *Prevotella* has many different species that could perform different actions. Some species have cellulolytic activity but were primarily involved in the fermentation of the hydrolysis products. With degradation of the biomass, hydrolysis and fermentation happened simultaneously, which could explain the fluctuation phenomena of *Prevotella* abundance (Xin et al., 2019). The abundance of *Rikenellaceae* increased from 2.55% to 11.45%, while the abundance of *Eubacterium* decreased from 9.47% to 0.70% with the digestion time. *Rikenellaceae* has been reported to play possible roles in the degradation of polysaccharides (Seshadri et al., 2018). The increase in *Rikenellaceae* could be related to the increased hydrolysis efficiency in the latter digestion time. All these findings indicated that the dynamic change of the microbial community is crucial in hydrolyzing the lignocellulose and utilizing the carbohydrates.

### 3.4 Hydrolysis of corn stover with rumen fluid collected at different digestion times

**FIGURE 3:**
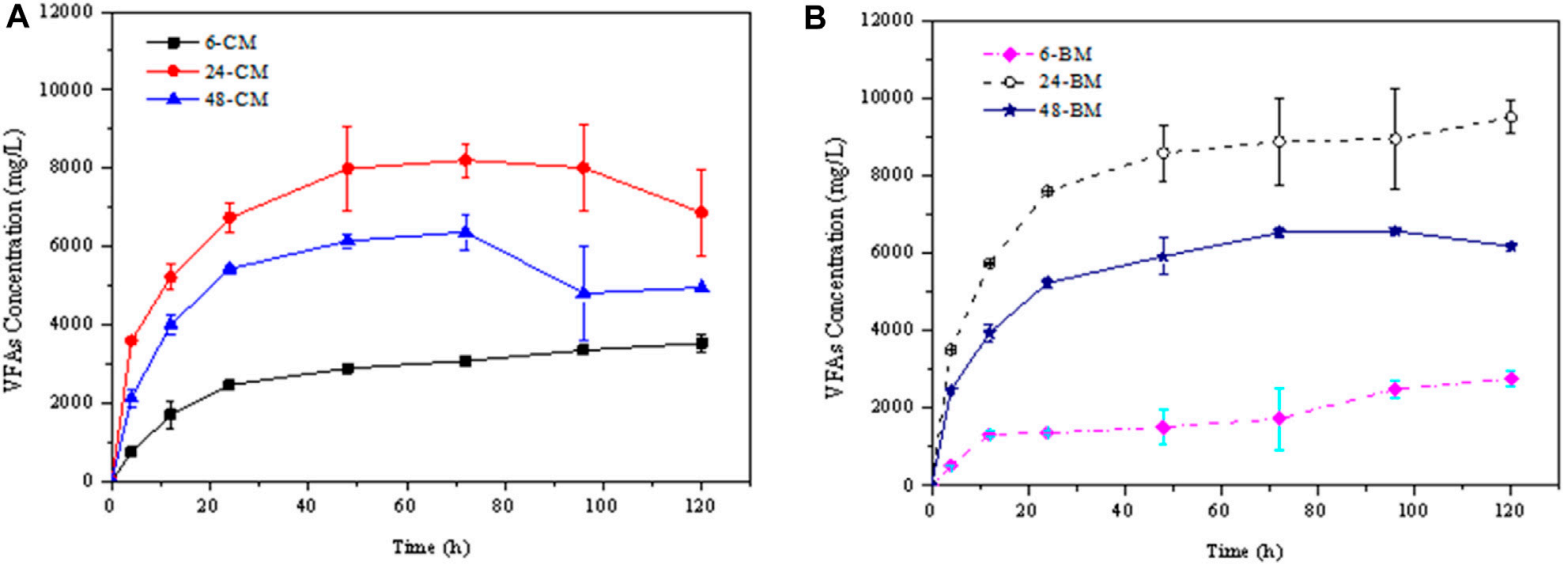
VFA production for the hydrolysis kinetics of the coarse milled (CM) and ball-milled (BM) corn stover treated with rumen fluid collected at different digestion times. **(A)** represented the VFA production of CM corn stover, **(B)** represented the VFA production of BM corn stover.

According to the composition of the microorganisms, rumen fluids collected at three different digestion times (6, 24 and 48 h) were chosen to perform the hydrolysis experiments. The hydrolysis characteristics were comparatively analyzed. Figure 3 shows the total net VFA concentrations during the hydrolysis kinetics of CM and BM corn stover which have deducted the VFA concentrations in the initial rumen fluid. In the hydrolysis of corn stover with rumen fluid, the dominant VFA is acetic acid, followed by propionic and butyric acids, consistent with the former rumen inoculum studies (Ramos et al., 2018; Nguyen et al., 2020). The dominant VFA concentrations after the hydrolysis for 120 h were shown in Table 3. The highest VFA concentrations and hydrolysis rates for CM and BM corn stover were achieved by treating with the rumen fluid collected at 24 h (respectively labelled as 24-CM and 24-BM). The CM and BM corn stover hydrolyzed with rumen fluid collected at 24 h produced approximately two- and three-folds higher VFA concentrations, respectively than that hydrolyzed with rumen fluid collected at 6 h (respectively labelled as 6-CM and 6-BM). The VFA production from the corn stover treated with rumen fluid collected at 48 h (respectively labelled as 48-CM and 48-BM) was intermediate between these productions.

**TABLE 3:** The dominant acid concentrations in the hydrolysis at 120 h.

Sample	Acetic acid (mg/L)	Propionic acid (mg/L)	Butyric acid (mg/L)	Total VFA (mg/L)
6-CM	3526.27 ± 408.86	8.05 ± 177.65	27.81 ± 396.76	3534.32 ± 231.21
6-BM	2756.07 ± 193.25	76.96 ± 53.44	0 ± 8.09	2756.07 ± 193.25
24-CM	5356.58 ± 880.28	1262.51 ± 326.94	249.03 ± 116.27	6868.12 ± 1090.95
24-BM	6784.68 ± 1024.50	2014.81 ± 101.45	708.41 ± 705.57	9507.89 ± 420.38
48-CM	1758.15 ± 1963.93	441.39 ± 807.57	876.55 ± 117.37	3076.09 ± 2654.13
48-BM	3844.13 ± 79.80	1374.76 ± 76.49	963.29 ± 15.39	6182.18 ± 12.08

Note: 6-CM, and 6-BM, represent the coarse milled (CM) and ball-milled (BM) corn stover treated with rumen fluid collected at 6 h; 24-CM, and 24-BM, represent the CM, and BM, corn stover treated with rumen fluid collected at 24 h; 48-CM, and 48-BM, represent the CM, and BM, corn stover treated with rumen fluid collected at 48 h, respectively.

Interestingly, we comparatively analyzed the main microbial proportion in the fluid (Table 4) and found that the microbial composition has great influence on the biodegradation of corn stover. For microbes at the phylum level, the proportion of *Bacteroidetes* to *Firmicutes* of the rumen fluid collected at 6 h is 0.78, largely lower than the other two rumen fluids, which is disadvantage to the hydrolysis. This indicated the proportion of *Bacteroidetes* provides an additional contribution to the hydrolysis. As to the microbes at the genus level, the proportion of *Prevotella*/*Rikenellaceae* shows negative relationship with the VFA production, indicating *Prevotella* play a crucial role in the fermentation.

**TABLE 4:** Main microbial proportion in the rumen fluid collected at different times.

Samples	6 h	24 h	48 h
Bacteroidetes/Firmicutes proportion	0.78	1.97	3.32
Prevotella/Rikenellaceae proportion	9.60	2.22	2.09

For the corn stover of different particle sizes, the hydrolysis products of BM samples treated with rumen fluid collected at 24 h and 48 h increased by approximately 38.42% and 24.84%, respectively than those of CM samples. However, the VFA production decreased by 28.23% for the hydrolysis of BM corn stover with rumen fluid collected at 6 h. Many scholars found ball milling as an efficient pretreatment method to increase the hydrolysis efficiency (Liu et al., 2020; Shen et al., 2020). However, the decrease in particle size of corn stover showed negative effect on the hydrolysis with rumen fluid collected at 6 h. Agglomeration phenomena could occur for the BM particles (Ji et al., 2016), which may affect the adsorption of microorganisms. The adsorption of *Firmicutes* may be easily influenced compared with *Bacteroidetes.* Thus the hydrolysis of 6-BM sample was lower than that of 6-CM sample.

After comprehensively comparing the VFA productions from two different scale corn stover treated with rumen fluid collected at different digestion times, it was found that the maximum VFA concentration of different scale corn stover was about twice (48-BM sample compared with the 48-CM sample). On the contrary, the VFA concentration of the same scale corn stover treated with rumen fluid collected at different times was about three times higher (24-BM sample compared with the 6-BM sample). These results indicated that the microbial community diversity seems to contribute more to the VFA production compared to the substrate structures which also proved the importance of understanding the essential function of rumen microbiome (Seshadri et al., 2018).

### 3.5 Fermentation of the corn stover with rumen fluid and exogeneous cellulase

**FIGURE 4:**
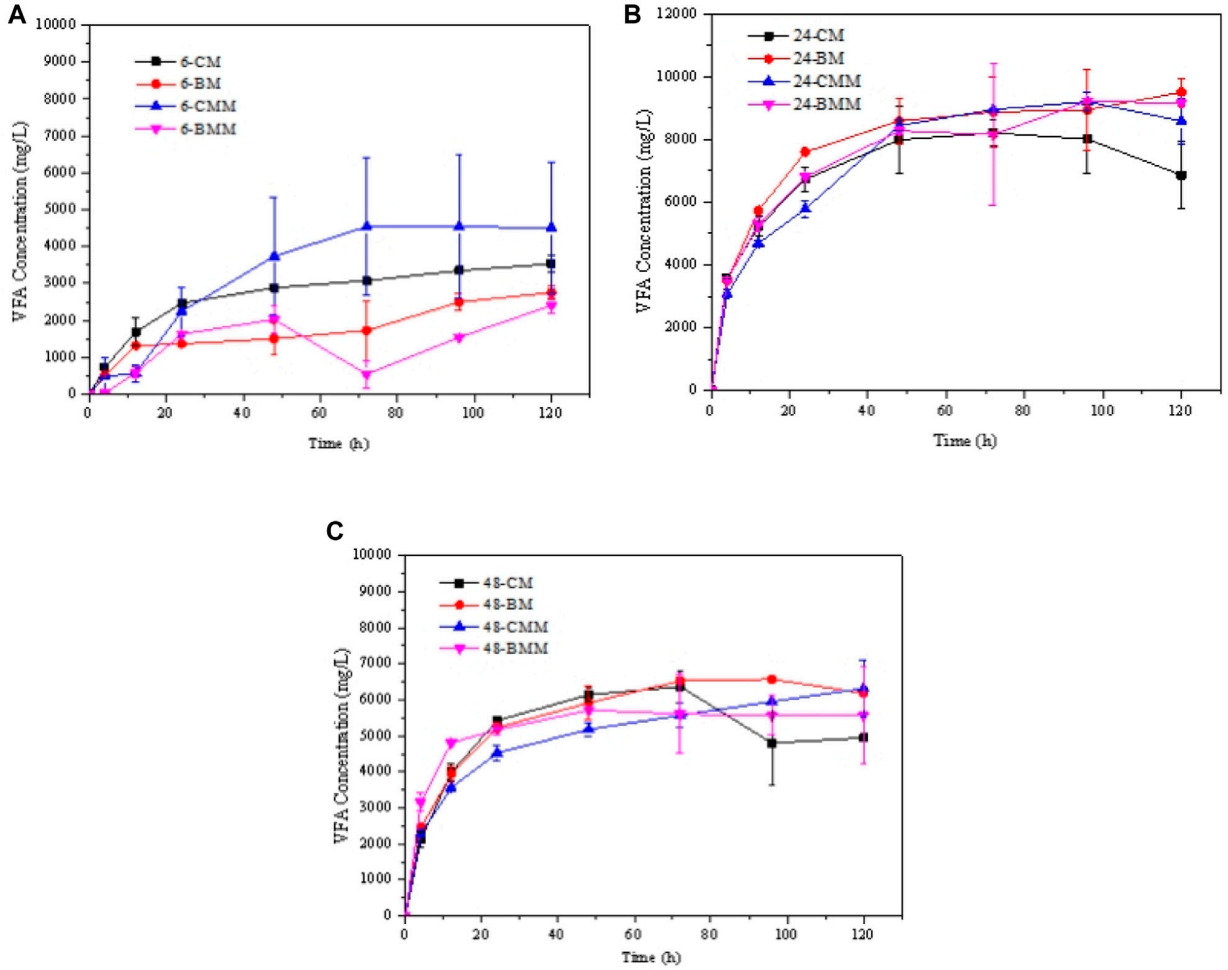
VFA production in the hydrolysis kinetics of coarse milled (CM) and ball-milled (BM) corn stover treated with rumen fluid collected at different times with or without exogeneous cellulase. **(A)** 6 h; **(B)** 24 h; **(C)** 48 h.

The total VFA concentrations during the ruminal hydrolysis kinetics with exogeneous cellulase of CM and BM corn stover are shown in Figure 4 (the samples hydrolyzed with cellulase were labelled as-CMM and-BMM, respectively). The VFA concentration treated with rumen fluid and exogenous cellulase seldom changed compared to the hydrolysis without adding exogenous cellulase. This is because some by-products that inhibit the cellulase activity may be produced during the ruminal fermentation thus limited the further hydrolysis. Besides, composition of the rumen microorganisms may also have a certain effect on cellulase activity. The enzymes secreted by different microorganisms may have synergistic promoting effects and inhibiting effects on exogenous cellulase (Devendran et al., 2016). Therefore, it is necessary to analyze the product compositions and the internal reasons further. In addition, the proper reaction conditions considering both the rumen microorganisms and the exogeneous cellulase should be further optimized to achieve high hydrolysis production.

**FIGURE 5:**
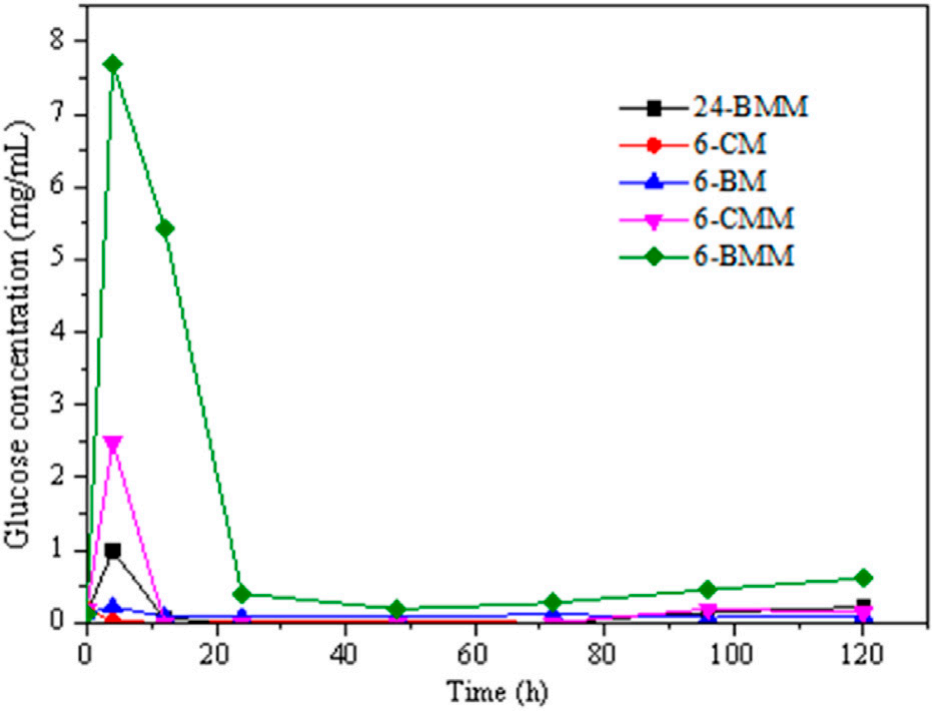
Glucose production in the hydrolysis of coarse milled (CM) and ball-milled (BM) corn stover treated with rumen fluid and exogeneous cellulase.

The glucose concentration in the ruminal fermentation with or without exogenous cellulase are shown in Figure 5. The results indicate that there was basically no glucose in the ruminal fermentation system. Only a small amount of glucose can be detected in the initial reaction stage which dropped significantly after 12 h of hydrolysis. This is because the glucose was quickly degraded and converted into VFA and other products via rumen microorganisms. Compared with other two treatments, the samples treated with the rumen fluid collected at 6 h produced the most glucose, which could be related to the difference in microbial community abundance. The rumen fluid collected at 6 h had a high abundance of *Prevotella* thus the number of microorganisms converting glucose into VFA would be relatively lower. Because *Prevotella* is the dominant cellulolytic genus which can convert more cellulose into glucose (Xin et al., 2019). Meanwhile, more glucose was stored in the hydrolysis system, which also demonstrated the lower VFA production of 6-CM and 6-BM samples. All these results indicated that rumen microbial species have an important effect on the degradation products. The samples with exogeneous cellulase produced more glucose in the initial stage, indicating that the cellulase also contributed to the production.

### 3.6 Correlation analysis of microbial abundance and ruminal hydrolysis

**FIGURE 6:**
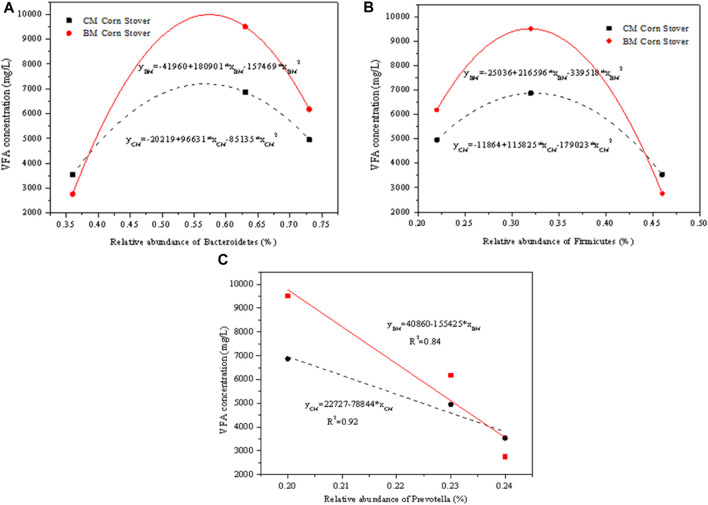
Correlation between the volatile fatty acids (VFA) concentration and the relative abundance of **(A)** *Bacteroidetes*, **(B)** *Firmicutes*, and **(C)** *Prevotella*.

The corn stover treated with rumen fluid collected at different digestion times produced different amounts of VFAs, largely dependent on the different microbial abundance in rumen fluid. Figure 6 shows the correlation between the VFA concentration and the relative abundance of the microorganisms. The *Bacteroidetes* and *Firmicutes* had an exponential relationship with the VFA concentration for CM and BM corn stover. This finding indicates that excessively high or low abundance of *Bacteroidetes* and *Firmicutes* are disadvantageous for the fermentation. The previous study also revealed the ecological and functional importance of *Bacteroidetes* and *Firmicutes* in ruminant (Xin et al., 2019). The dominant genus *Prevotella* showed a negative linear relationship with the VFA concentration within a small abundance range. Even through *Prevotella* is the most abundant genus of microorganisms and is reported essential to the fermentation, high abundance could not lead to high VFA production. As a result, it should be noticed that *Prevotella* was not the decisive genus, and it should cooperate with other microorganisms synergistically to obtain the optimum production (Devendran et al., 2016). In the later studies, additional attention should be paid to develop multi-fold cooperative microbials other than single microorganisms to improve the hydrolysis and fermentation. The correlation analysis quantitatively clarified the dependency mechanism of VFA production on rumen microorganisms and could further improve the understanding of applying the microbiota in ruminants to degrade the biomass and produce the VFA.

## 4 Conclusion

The VFA production of BM corn stover was higher than that of CM corn stover due to its accessible structural properties (smaller particle size, lower cellulose crystallinity and larger pore size). The abundance levels of *Bacteroidetes* increased from 29.98% to 72.74%, and that of *Firmicutes* decreased from 51.76% to 22.11% with the prolonged digestion time. The corn stover treated with the rumen fluid collected at 24 h achieved the highest VFA production because the proportion of the *Bacteroidetes* and *Prevotella* provide an additional contribution to the fermentation. The *Bacteroidetes* and *Firmicutes* showed an exponential relationship with the VFA concentration. *Prevotella* was the most abundant genus and showed linear relation with the VFA concentration. The microbial community abundance contributed more to the VFA production compared with the substrate structures.

## Data Availability

The original contributions presented in the study are publicly available. This data can be found here: https://datadryad.org/stash/landing/show?id=doi%3A10.5061%2Fdryad.z34tmpgm5.
